# The Role of Diabetes and Hyperglycemia on COVID-19 Infection Course—A Narrative Review

**DOI:** 10.3389/fcdhc.2022.812134

**Published:** 2022-03-10

**Authors:** Evangelia Tzeravini, Eleftherios Stratigakos, Chris Siafarikas, Anastasios Tentolouris, Nikolaos Tentolouris

**Affiliations:** ^1^ First Department of Propaedeutic Internal Medicine, Medical School, National and Kapodistrian University of Athens, Laiko General Hospital, Athens, Greece; ^2^ Gastroenterogy Department, Athens General Hospital “G Gennimatas”, Athens, Greece

**Keywords:** diabetes mellitus, hyperglycemia, COVID-19, outcome, post-discharge

## Abstract

It was previously reported that subjects with diabetes mellitus (DM) are more vulnerable to several bacterial or viral infections. In the era of coronavirus disease 2019 (COVID-19) pandemic, it is reasonable to wonder whether DM is a risk factor for COVID-19 infection, too. It is not yet clear whether DM increases the risk for contracting COVID-19 infection or not. However, patients with DM when infected are more likely to develop severe or even fatal COVID-19 disease course than patients without DM. Certain characteristics of DM patients may also deteriorate prognosis. On the other hand, hyperglycemia per se is related to unfavorable outcomes, and the risk may be higher for COVID-19 subjects without pre-existing DM. In addition, individuals with DM may experience prolonged symptoms, need readmission, or develop complications such as mucormycosis long after recovery from COVID-19; close follow-up is hence necessary in some selected cases. We here present a narrative review of the literature in order to set light into the relationship between COVID-19 infection and DM/hyperglycemia.

## 1 Introduction

The coronavirus disease 2019 (COVID-19) caused by severe acute respiratory syndrome coronavirus 2 (SARS-CoV-2) has evolved into a world pandemic with almost 5 million deaths so far (1, 2). On the other hand, diabetes mellitus (DM) is also a global burden; the International Diabetes Federation estimated that in 2019, one in 11 adults had DM, which is translated to 463 million people worldwide (3). We could therefore say that DM is a pandemic itself. Previously, DM was also identified as an independent risk factor for severe disease from two other members of the coronavirus family, SARS and Middle East Respiratory Syndrome (MERS) (4, 5). It is hence reasonable to assume that DM may affect the COVID-19 infection course.

Investigation of the way SARS-CoV-2 affects host cells may enlighten the reasons for increased vulnerability of patients with DM to COVID-19 infection. After inhalation, the first step for SARS-CoV-2 invention into host cells is binding to angiotensin-converting enzyme 2 (ACE-2) receptor on cell membrane (6). Receptors of ACE-2 are found on epithelial cells of the lungs but are also expressed on several other tissues, such as the heart, vessels, pancreas, and kidneys (7). Infection by SARS-CoV-2 can therefore damage several organs beyond the lungs (7). Some studies support that lungs of patients with DM overexpress ACE-2 receptors (8), either due to DM alone or as a result of pharmacological treatment (9); patients with DM could therefore be more vulnerable to SARS-CoV-2 entry into the host cells. Regardless the ACE-2 receptor expression, hyperglycemia may also increase SARS-CoV-2 cell entry through glycosylation of ACE-2 receptors (10, 11).

Moreover, COVID-19 infection is linked to a profound inflammation and release of cytokines such as intereukin-6 (IL-6) and tumor necrosis factor-a (TNF-a), which can lead to the so-called cytokine storm and severe disease (11, 12). Diabetes mellitus is also characterized by a chronic, pro-inflammatory state, so patients with DM are more likely to develop a catastrophic inflammatory response to COVID-19 infection (13, 14). Excess cytokine circulation in turn increases insulin resistance (15). On the other hand, b-cell also expresses ACE-2 receptors and can be infected by SARS-CoV-2, with b-cell damage and apoptosis as a consequence (16). The reduction in insulin production can then accelerate pre-existing DM and induce hyperglycemia or even new onset diabetes in previously normoglycemic subjects (16). Hence, a vicious cycle between COVID-19 and hyperglycemia may evolve.

In addition, another mediator between COVID-19 infection and hyperglycemia/DM might be oxidative stress; excess free radicals seem to promote viral entry into the cells and severe tissue damage (17). Hyperglycemic states, in turn, enhance oxidative stress (18), and they can consequently increase the risk for severe COVID-19 infection. Finally, hyperglycemia directly and indirectly through inflammation and oxidative stress promotes endothelial damage and hypercoagulation, two key mechanisms in COVID-19 infection course (12, 19).

The relationship between COVID-19 and DM may therefore be bidirectional; the presence of DM enhances SARS-CoV-2 catastrophic action, and SARS-CoV-2 in turn induces hyperglycemia and insulin resistance.

We here conducted a review of the literature in order to (1) investigate the role of DM on COVID-19 disease progression, (2) explore the importance of hyperglycemia for COVID-19 infection prognosis, (3) address patients characteristics that may increase the risk of unfavorable outcomes, and (4) highlight possible post-discharge implications.

For the purpose of this review, we researched PubMed database using keywords “diabetes mellitus and COVID-19”, “hyperglycemia and COVID-19”, and “glucose and COVID-19”. We included articles that were published until September 15, 2021, all in the English language. We excluded articles that seemed irrelevant based on their title, or abstract, or after reading the main body of the text. We also searched reference lists of the included articles. We then composed a narrative review of the gathered literature.

## 2 DM as a Risk Factor for COVID-19 Infection

Patients with DM seem to be more prone to several bacterial and viral infections than the general population (20). However, it is not yet clear whether the presence of DM increases the risk for contracting COVID-19 infection. In a large web-based survey with 780,961 participants, DM was an independent risk factor for COVID-19 infection, with an odds ratio (OR) of 1.46 [95% confidence interval (CI), 1.23–1.74] (21). Likewise, among 219,729 individuals that were examined by Park et al., patients with DM experienced a higher risk for COVID-19-positive test than subjects without DM (OR, 1.15; 95% CI, 1.07–1.24) (22). Crouse et al. also reported a twofold risk of contracting COVID-19 infection for DM subjects (OR, 2.11; 95% CI, 1.78–2.48) (23). In addition, Gutierrez et al. observed that the prevalence of DM was higher among individuals who tested positive for COVID-19 than those with a negative result (16.2% versus 10.1%, respectively) (24). Comparable were the findings of another large study with about half a million participants, which recorded DM in 16.6% of COVID-19-positive and only in 9.5% of COVID-19-negative individuals (25). Three research groups from Mexico also reported DM prevalence from 16.1% to 18.4% among COVID-19-positive inpatients and outpatients (26–29); this was higher than the prevalence of DM in the general population of Mexico, which, in 2016, was estimated at 9.4% (30). Likewise, in a large study from the USA, DM was the second most frequent comorbidity, corresponding to 15% of COVID-19-infected individuals, while the prevalence of DM in the same region was 9.7% (31).

On the other hand, in a study of 10,069 COVID-19-infected patients and 50,587 matched controls, patients with DM tented to be more susceptible to COVID-19 infection, but the risk was not statistically significant (OR, 1.06; 95% CI; 0.97–1.16) (32). In the subgroup analysis, however, DM patients on insulin use, and especially men and those aged 40-59 years old, were more likely to be infected than participants without DM (32). On the contrary, in a meta-analysis of 41 studies from China, the pooled prevalence of DM was lower among COVID-19-positive individuals compared with the general population (9% versus 10.9%, respectively, p < 0.0001) (33). Another group of investigators also observed that DM patients corresponded to 8.8% of COVID-19-positive individuals (34). Similarly, a meta-analysis of 212 studies from 11 countries reported a DM prevalence of 10.2% among COVID-19 positive subjects (35), comparable with the worldwide prevalence of DM (36).

Based on the above findings, the relationship between DM and the risk for COVID-19 infection is not well documented. The heterogeneity in the design of the aforementioned studies, and differences in local policy for COVID-19 spread prevention, may be responsible for the lack of consensus.

## 3 DM and COVID-19 Infection Outcomes

### 3.1 DM as a Risk Factor for Hospitalization From COVID-19 Infection

The presence of DM seems to increase the risk for hospitalization among COVID-19 infected individuals (24, 37). In a study based on 211,003 medical records of COVID-19 infected subjects, patients with DM had an almost twofold greater risk for hospital admission (38). Similar were the findings of Gottlieb et al. and Martos-Benirez et al. (39, 40). In a large cross-sectional study from Mexico as well, COVID-19-positive patients with self-reported DM had 38.4% (95% CI, 37.6–39.2) predicted probability for hospitalization; the estimated probability was higher when one or more comorbidities were added to DM (28). Halalu et al., on the other hand, in a small multicenter study, reported a significantly increased hospitalization rate not only for DM but also for prediabetes (41). In addition, according to a study from the USA with almost a million participants, 20.5% of COVID-19 hospitalizations were attributable to DM; investigators also estimated that a 10% reduction in DM prevalence could reduce hospital admissions for COVID-19 infection by 2.7% (42). From another point of view, the prevalence of DM among COVID-19-positive hospitalized patients ranged from 19.4% to 33.8% (43, 44), which was much higher than the prevalence of DM in the corresponding general populations (9.7% and 8.9% for USA and Europe respectively) (3, 31). On the contrary, according to another study, DM was not an independent risk factor for hospitalization (45). Certain characteristics of DM patients may increase the risk of hospitalization after COVID-19 infection; COVID-19-positive patients with DM and one or more comorbidities, such as obesity, hypertension, chronic kidney disease, or chronic obstructive pulmonary disease were more likely to need hospital admission than COVID-19-infected subjects with DM alone in several studies (24, 28, 37, 46). Of note, however, Maddaloni et al. reported that cardiovascular disease was not an independent risk factor for hospitalization among patients with DM, perhaps due to overlap in pathophysiology of the two diseases (46). Apart from comorbidities, older age, male sex, delayed search of medical help, and exact time point during the pandemic increased the risk of hospitalization among individuals with DM (28, 37). In addition, socioeconomic factors such as living in a community with meager health resources or social isolation, indigenous language speaking, and the type of health care provider also influenced the chance of admission for COVID-19-infected patients with DM (28). Nevertheless, some of the above factors may truly increase vulnerability of individuals with DM to severe COVID-19 infection requiring hospital admission, while others are rather linked to doctors’ perception and influence their decisions.

### 3.2 DM as a Risk Factor for Severe COVID-19 Disease

#### 3.2.1 DM as a Risk Factor for ICU Admission

Several data highlight DM as a risk factor for severe COVID-19 infection. The presence of DM increased the risk for intensive care unit (ICU) admission in a small study by Abohamr et al. (47). According to another study from Mexico, DM independently increased the risk for severe COVID-19 infection on admission, defined as pneumonia or organ failure that requires treatment in the ICU (48). Similar were the results from a large study based on over 200,000 medical records of COVID-19-infected subjects, with a 66% higher risk for ICU admission among DM patients (38). Data from 1,157 patients hospitalized in two London hospitals also supported that DM is a risk factor for critical care requirement (49); the combined outcome of critical care or death did not, however, reach statistical significance (49).

#### 3.2.2 DM as a Risk Factor for Mechanical Ventilation

In addition, the need for mechanical ventilation was reported to be higher among DM patients with COVID-19 infection compared with those without DM (50). The presence of DM increased the risk of intubation in COVID-19-infected patients with a slightly higher OR when obesity or obesity and hypertension accompanied DM (24). In the study by Hernandez-Galdamez et al. as well, DM was an independent risk factor for endotracheal intubation (38). Similar were the results reported by Moon et al.; DM was independently associated with the need for supplemented oxygen or ventilator support, even after adjustment for age, sex, and comorbidities (45).

#### 3.2.3 DM as a Risk Factor for Complicated Disease Course

Moreover, in a study based on national electronic health records, DM increased the risk for COVID-19 pneumonia, ICU admission, and intubation in the multivariable analysis compared with individuals without DM (40). Jakob et al. also observed that DM was an independent risk factor for a complicated clinical picture on the day of COVID-19 infection diagnosis (51). Likewise, Huang et al. suggested that patients with DM were more likely to have severe (defined as respiratory rate >30 breaths/min, blood oxygen saturation ≤93%, oxygenation, index ≤300 mmHg, and/or lung infiltrates increased >50% within 24–48 h) or critical (defined as respiratory failure, septic shock, and/or multiple organ dysfunction/failure) COVID-19 infection than patients without DM (52). According to another study with 5,685 COVID-19 patients, DM also increased the risk for severe disease course (53), with severity being defined using World Health Organization criteria (54). In addition, results from a Spanish cohort highlighted DM as a risk factor for acute respiratory distress syndrome (ARDS) among COVID-19-infected patients (55). Based on data from Wuhan, patients with DM were also more likely to develop complications such as ARDS, acute kidney injury (AKI), acute cardiac injury (ACI), septic shock, or secondary infections and need ICU admission or non-invasive mechanical ventilation; however, the risk of death and the need of mechanical ventilation were not significantly higher among DM patients in this cohort (56).

Chen et al. examined patients with type 2 DM (T2DM) and observed significantly more complications from COVID-19 infection, namely, ARDS, AKI, ACI, coagulopathy, and hypoproteinemia, compared with patients without DM (57). Type 2 DM was also reported as an independent risk factor for ICU admission, after adjustment for age, sex, comorbidities, and insurance status (58). Moreover, in a large cohort of COVID-19 hospitalized participants, patients with T2DM had higher rates of ICU admission and intubation, and longer hospital stay compared with patients without DM (59). Similar were the results reported by Sun et al.; T2DM was independently associated with the risk for ADRS and severe disease (defined as respiration rate ≥30 times/min, blood oxygen saturation ≤93%, oxygenation index ≤300 mmHg, organ failure that requires ICU or shock) (60).

The results of the aforementioned studies are presented in summary in **Table 1**.

**Table 1:** Studies that highlight diabetes mellitus as a risk factor for mortality and/or complications from COVID-19 disease.

First Author(ref)	Study population	Health structure	Region	N	OR HR [95% CI]**		p	Outcome
Abohamr et al. (47)	Record-based case-series study	Inpatients and outpatients	Saudi Arabia	768	OR 2.29 [1.70–3.06]		0.001	ICU admission
					OR 3.01, [1.87–4.84]			Death
Denova-Gutierrez et al. (48)	Cross-sectional, electronic record nationwide study*	Inpatients and outpatients	Mexico	23,593	aOR 1.87, [1.41–4.26]		–	ICU admission
Hernandez-Galdamez (38)	Cross-sectional, electronic record nationwide study	Inpatients and outpatients	Mexico	212,802	aOR 1.98, [1.93–2.03]		< 0.001	Hospitalization
					aOR 1.66, [1.56–1.77]			ICU admission
					aOR 1.68, [1.58–1.78]			Intubation
					aOR1.69, [1.63–1.74]			Death
Galloway et al. (49)	Two center observational study	Inpatients	England	1,157	HR 1.42, [1.04–1.95]		0.029	Critical Care
					HR 1.24, [0.95–1.60]		0.109	Death
					HR 1.20, [0.97, 1.48]		0.092	Death or Critical Care
Capak et al. (50)	Cross-sectional, electronic record nationwide study	Inpatients and outpatients	Croatia	2,249	–		< 0.001	Mechanical Ventilation
Gutierrez and Bertozzi (24)	Cross-sectional, electronic record nationwide study*	Inpatients and outpatients	Mexico	1,378,002	OR 2.28, [2.22–2.34]		< 0.01	Hospitalization
					OR 1.10 [1.05–1.15]			Intubation
					OR 1.82, [1.76–1.88]			Death
Moon et al. (45)	Nationwide population-based cohort study	Inpatients and outpatients	South Korea	5,307	aOR1.07, [0.72–1.59]		0.735	Hospitalization
					aOR 1.90, [1.28–2.92]		<0.001	Ventilator Support
					aOR2.66, [1.90–3.73]		<0.001	Death
Martos-Benitez et al. (40)	Retrospective, electronic record nationwide study*	Inpatients and outpatients	Mexico	38,324	aOR 2.11, [1.94–2.28]		<0.001	Hospitalization
					aOR 1.80, [1.66–1.95]			Pneumonia
					aOR 1.43, [1.21–1.48]			ICU admission
					aOR 1.46, [1.25–1.72]			Mechanical Ventilation
Gottlieb et al. (39)	Retrospective, registry-based cohort study	Inpatients and outpatients	USA	8,673	aOR 1.85, [1.53–2.22]		–	Hospitalization
					aOR1.21, [0.93 – 1.58]			Critical Illness
Jakob et al. (51)	Electronic record international, multicenter study	Inpatients and outpatients	Europe	2,155	aOR 1.33, [1.04–1.69]		0.023	Complicated Clinical Stage
Al Kuwari et al. (53)	Case series	Inpatients and outpatients	Qatar	5,685	OR 3.17, [1.76– 5.71]		0.0001	Severe/ Critical Disease
Shi et al. (56)	Two-center retrospective study	Inpatients	China	1,561	–		<0.05	ARDS
					–			AKI
					–			ACI
					–			Shock
					–			ICU admission
					aHR 1.58, [0.84–2.99]		>0.05	In hospital mortality
Rodriguez-Gonzalez et al. (55)	Retrospective, single center study	Inpatients	Spain	1,255	aOR1.93, [1.34–2.78]		<0.001	ARDS
					aOR1.45, [1.09–1.92]			Death
Chen et al. (57)	Retrospective, single center study	Inpatients	China	1,105	aHR 1.37, [1.03–1.81]		0.029	ARDS
					aHR1.76, [1.14–2.71]		0.010	AKI
					aHR 1.50, [1.05–2.15]		0.025	ACI
					aHR1.58, [1.03–2.44]		0.036	Coagulopathy
					aHR 1.47, [1.04–2.08]		0.028	Death
You et al. (58)	Retrospective, electronic record nationwide study	Inpatients and outpatients	Korea	5,473	aOR 1.59, [1.02–2.49]		0.042	ICU admission
					aOR1.90, [1.13–3.21]		0.016	In hospital mortality
Sonmez et al. (59)	Retrospective, electronic record nationwide study	Inpatients	Turkey	18,426	–		<0.001	Intubation
					–			ICU admission
					HR 1.75, [1.58–1.93]			In hospital mortality
Sun et al. (60)	Retrospective cohort study	Inpatients	China	3,400	aOR 4.38, [2.41–7.95]		<0.001	ARDS
					aOR 2.21, [1.60, 3.06]			Severe Disease
					aOR 5.26, [2.39–11.58]			In hospital mortality
Kim et al. (61)	Retrospective, electronic record nationwide study	Inpatients and outpatients	Korea	7,590	HR1.87, [1.41–2.48]		<0.0001	Mortality
Carrilo-Vega et al. (37)	Retrospective, electronic record nationwide study	Inpatients and outpatients	Mexico	10,544	OR 2.14, [1.70–2.69]		<0.001	Hospitalization
					OR 1.50, [1.13–1.98]		0.005	Mortality
Najera and Ortega-Avila (62)	Retrospective, electronic record nationwide study	Inpatients and outpatients	Mexico	515,090	OR 1.63, [1.66–1.59]		–	Mortality
De Souza et al. (63)	Cross-sectional observational study	Inpatients and outpatients	Brazil	9,807	aOR 2.33, [1.99–2.74]		<0.0001	Mortality
Williamson et al. (64)	Retrospective, electronic record nationwide study	Inpatients and outpatients	England	17,278,392	HbA1c < 58 mmol/mol	aHR 1.31, [1.24–1.37]	–	Mortality
					HbA1c ≥ 58 mmol/mol	aHR 1.95, [1.83–2.08]		
					no recent HbA1c	aHR 1.90, [1.72–2.09]		
Parra-Bracamonte et al. (26)	Retrospective, electronic record nationwide study	Inpatients and outpatients	Mexico	331,298	aOR 1.30, [1.24–1.34]		<0.001	Mortality
Zhu et al. (65)	Retrospective, multi-centered study	Inpatients	China	7,337	aHR 1.49, [1.13–1.96]		0.005	In-hospital mortality
Silva et al. (66)	Retrospective, multi-centered study	Inpatients	Brazil	120,804	aHR 1.14, [1.11–1.18]		–	In-hospital mortality
Shin et al. (67)	Nationwide case series study	Inpatients	Korea	5,771	aOR 2.26, [1.46–3.49]		<0.01	In-hospital mortality
Esme et al. (68)	Retrospective, electronic record nationwide study	Inpatients, Elderly	Turkey	16,942	60–79 years	aOR 1.18, [1.06–1.30]	0.001	In-hospital mortality
					≥80 years	aOR 1.26, [1.07–1.49]	0.006	

N, number of participants; OR, odds ratio; HR, hazard ratio; 95% CI, 95% confidence interval; aOR, adjusted OR (in the multivariable analysis); aHR, adjusted HR (in the multivariable analysis); ICU, Intensive Care Unit; T2DM, type 2 diabetes mellitus; ARDS, acute respiratory distress syndrome; AKI, acute kidney injury; ACI, acute cardiac injury; USA, United States of America; HbA1c, glycated hemoglobin.

*Studies that included both SARS-CoV-2-positive and SARS-CoV-2-negative individuals.

**Risk for SARS-CoV-2-positive patients with diabetes mellitus compared with SARS-CoV-2-positive patients without diabetes.

#### 3.2.4 DM as a Risk Factor for Severe COVID-19 Infection: Evidence From Meta-analysis

The results of several meta-analyses are in concordance with the aforementioned studies. In a meta-analysis of 61 studies, the presence of DM almost doubled the risk for severe COVID-19 disease, ICU admission, and invasive ventilation and tripled the risk for ARDS and progression to severe disease (69). Similar were the results of Huang et al., with the exception of ICU admission that did not reach statistical significance; of interest, the relative risk for severe COVID-19 infection was higher among younger patients (70). On the contrary, two other study groups observed a significant association between DM and ICU admission (71, 72). In addition, Kumar et al. estimated a triple risk for severe disease among patients with DM, after the analysis of 58 studies (73). Four more resent meta-analyses also reported a statistically significant risk for severe COVID-19 disease among DM patients (74–77). Moreover, Liang et al. highlighted DM as an independent predictor of disease severity (78).

**Table 2** summarizes the main findings of the aforementioned meta-analyses.

**Table 2:** Meta-analyses that highlight diabetes mellitus as a risk factor for mortality and/or complications from COVID-19 disease.

First Author (ref)	N of Studies	N of participants	Risk, 95% CI	p	Heterogeneity (I2%)	Outcome
Fang et al. (69)	61	~10,000	RR 1.95, [1.60–2.36]	<0.001	42.6	Severe Disease
			RR 3.30, [1.08–10.07]	0.036	0.0	Progression to Severe Disease
			RR 1.88, [1.10–3.23]	0.021	51.4	ICU admission
			RR 3.07, [1.28–7.36]	0.012	62.9	ARDS
			RR 1.85, [1.24–2.76]	0.003	50.8	Invasive Ventilation
			RR 1.75, [1.27–2.41]	<0.001	67.1	Death
Huang et al. (70)	30	6,452	RR 3.31, [1.08–10.14]	0.04	0.0	Disease Progression
			RR 4.64, [1.86–11.58]	0.001	9.0	ARDS
			RR 1.47, [0.38–5.67]	0.07	63.0	ICU admission
			RR 2.45 [1.79–3.35]	<0.001	45.0	Severe Disease
			RR 2.12, [1.44–3.11]	<0.001	72.0	Death
Hu et al. (71)	30	6,685	Pooled OR 2.57, [1.91–3.46]	<0.001	36.1	Severe Disease
			Pooled OR 1.61, [1.04– 1.04]	0.032	76.8	ICU admission
Hussain et al. (72)	43	23,007	Pooled Risk Ratio 1.88, [1.20–2.93]	0.006	75.0	ICU admission
			Pooled Risk Ratio 1.61, [1.16–2.25]	0.005	93.0	Death
Kumar et al. (73)	58	6,892	Pooled OR 3.11, [1.99–4.88]	<0.05	48.0	Severe Disease
Li X et al. (74)	41	21,060	OR 2.40, [1.98–2.91]	–	55.6	Severe Disease
Giri et al. (75)	41	16,495	OR 2.04, [1.67–2.50]	<0.0001	55.0	Severe Disease
Honardoost et al. (76)	28	6,270	OR 2.61, [2.02–3.30]	–	30.5	Severe Disease
Nget al. (77)	4	2464	HR 1.94, [1.54–2.46]	–	0.0	Death
Liang et al. (78)	23	22,359	aHR1.61, [1.28–2.04] & aOR 1.58, [1.07–2.32]	–	58.2 & 29.2	Severe Disease
Li et al. (35)	212	281,461	Coefficient 23.4, [15.0–31.7]	<0.001	NA	Severe Disease
			Coefficient 8.2, [2.4–14.0]	0.006		Death
Kaminska et al. (79)	19	7,327	OR 2.38, [1.80–3.13]	<0.001	40.0	ARDS
			OR 1.97, [1.36–2.85]	<0.001	0.0	AKI
			OR 2.59, [1.81–3.73]	<0.001	57.0	ACI
			OR 1.43, [0.82–2.50]	0.20	85.0	Severe Disease
			OR 2.39, [1.65–3.64]	<0.001	62.0	Death
Wu et al. (80)	9	926	Pooled OR 1.75, [1.31–2.36]	0.0002	5.0	Death
Varikasuvu et al. (81)	47	13,268	OR 2.20, [1.69–2.86]	<0.00001	58.0	Severe Disease
			OR 2.52, [1.93–3.30]		31.0	Death
Palaiodimos et al. (82)	14	18,506	OR 1.65, [1.35–1.96]	–	77.4	Death
Silverio et al. (83)	45	18,300	Adjusted coefficient 1.02, [1.01–1.05]	<0.001	97	In hospital Mortality
Mantovani et al. (84)	83	78,874	OR 2.10, [1.71–2.57]	–	41.5	Severe Disease
			OR 2.68, [2.09–3.44]		46.7	Death

N, number of; OR, odds ratio; HR, hazard ratio; RR, relative risk; 95% CI, 95% confidence interval; aOR, adjusted OR (in the multivariable analysis); aHR, adjusted HR (in the multivariable analysis); ICU, intensive care unit; ARDS, acute respiratory distress syndrome; AKI, acute kidney injury; ACI, acute cardiac injury.

#### 3.2.5 DM as a Risk Factor for Severe Disease: Conflicting Data

There are, however, some evidence that does not support the relationship between DM and severe COVID-19 disease. In a study by Hasani et al., DM was not an independent risk factor for ICU admission or mechanical ventilation in the multivariable analysis (85). Kim et al. also reported DM as a risk factor for ICU admission, but not for severe disease overall [defined as non-invasive ventilation, invasive ventilation, extracorporeal membrane oxygenation (ECMO), and death] or for progression to severe disease (86). According to data from a large New York City cohort, DM increased the risk for critical illness (defined as a composite of ICU admission, need of mechanical ventilation, discharge to hospice, or death) among COVID-19-infected patients, but the relationship was attenuated in the fully adjusted model (87). Similar were the results of another study from Korea; DM was not an independent predictor of severe disease (defined according to oxygen needs, multiorgan failure/ECMO, and death) after adjustment for age, sex, and comorbidities (OR, 1.08; 0.82–1.42) (67). Tchang et al. also observed a higher incidence of the composite outcome (ICU admission, invasive mechanical ventilation, or in-hospital death), but the risk for ICU admission or invasive ventilation alone was not significantly higher in patients with DM (88). Lastly, in two meta-analysis, DM was a predictor of ICU admission, COVID-19 complications, and length of hospitalization but not of severe COVID-19 disease as a composite outcome (79, 89). Overall, patients with DM are more likely to face a complicated clinical course of COVID-19 infection than patients without DM. Only little evidence opposes this observation; the heterogeneity in the definition of severe COVID-19 disease in different studies, and over-adjustment for confounding factors in some others, may explain the lack of complete consensus.

### 3.3 DM as a Risk Factor for Mortality From COVID-19 Infection

#### 3.3.1 DM as a Risk Factor for Overall Mortality

With regard to mortality alone, DM patients have at least a twofold greater risk of dying from COVID-19 compared with individuals without DM. Two large studies, one from Mexico and one from Korea, based on outpatients and inpatients data, observed an increased risk of death in DM COVID-19-positive patients compared with COVID-19 subjects without DM (24, 61). Slightly lower ORs for DM but still statistically significant were reported by Carrilo-Vega et al., Hernandez-Galdamez et al., and Najera et al. all based on both inpatients and outpatients records from national databases (37, 38, 62). Another group also identified T2DM as a risk factor for death among COVID-19-positive patients; the risk was lower after adjustment for age, sex, and comorbidities, but remained significant, supporting that DM is an independent risk factor for mortality among COVID-19-infected individuals (26). In addition, among 44,672 COVID-19 subjects examined by Asfahan et al., older age and comorbidities increased mortality, with DM patients having a case fatality rate of 7.3% (90). In the OpenSAFELY, a huge population based study from England, investigators analyzed over 17 million electronic health records and reported DM as an independent risk factor for death from COVID-19 infection (64). Likewise, according to another population-based study focused on older adults (age ≥60 years old), the presence of DM significantly increased mortality from COVID-19 in the multivariable analysis (63).

#### 3.3.2 DM as a Risk Factor for In-Hospital Mortality

Zhu et al. reported higher in-hospital mortality for T2DM patients compared with non-DM (65). Additionally, in a large cohort from Brazil, hospitalized COVID-19-infected patients with DM were more likely to die than patients without DM (66). According to a study from Korea, DM independently associated with mortality in hospitalized patients with COVID-19 infection (67). Evidence from Turkey as well highlights DM as a risk factor for death in COVID-19-infected hospitalized patients in general (59) and in the elderly (68).

The main studies that examined the relationship between DM and COVID-19 mortality overall and in-hospital are shown in **Table 1**.

#### 3.3.3 Age and Comorbidities as Risk Factors for Mortality Among COVID-19-Infected DM Patients

A study with ~340,000 COVID-19-positive participants also reported a reverse relationship between risk of death and age among patients with DM (91). Likewise, Woolcott et al. assessed over 1.5 million medical records of COVID-19-positive and COVID-19-negative inpatients and outpatients and observed a stronger association between DM and mortality among younger COVID-19-infected patients; among subjects older than 80 years, DM was no longer a significant risk factor for death from COVID-19 [adjusted hazard ratio (aHR), 1.52; 95% CI, 1.40–1.66 for patients 20–39 years; aHR, 1.03; 95% CI, 0.98–1.08 for patients over 80 years old] (92). In addition, among DM patients with COVID-19 infection, the risk was higher for outpatients and female patients (92).

In another study from Mexico, however, DM was an independent risk factor for poor outcome for both inpatients and outpatients with COVID-19 infection, but the risk was higher for those admitted to hospital (aHR, 2.09; 95% CI, 1.09–4.00 for in-patients and aHR, 1.25; 95% CI, 1.02–1.53 for outpatients) (27). According to findings of Pena et al., on the other hand, although DM increased in-hospital mortality [adjusted OR (aOR), 1.19; 95% CI, 1.15–1.24], it was not independently associated with the risk of death among outpatients with COVID-19 infection (aOR, 1.09; 95% CI, 0.9–1.13) (34). Nevertheless, the combination of DM with obesity and/or arterial hypertension deteriorated prognosis significantly for both inpatients and outpatients (27, 34).

#### 3.3.4 DM as a Risk Factor for Mortality: Data From Meta-analysis

The results of several meta-analyses support the above findings (35, 79). Wu et al. estimated a pooled OR of 1.75 for mortality among DM patients with COVID-19 (80). Similar were the findings of a meta-analysis by Varikasuvu et al. and by Palaiodimos et al. (81, 82, 93). Of note, Corona et al., in a meta-analysis of 87 studies, also identified DM as the best predictor of dying from COVID-19 infection, and the association between DM and mortality was independent of sex but was weaker among older subjects (93). On the contrary, in other meta-analyses, the presence of DM seems to increase COVID-19 in hospital mortality, even after adjustment for confounding factors, age included (83, 84).

The results of the above meta-analyses are depicted in **Table 2**.

#### 3.3.1 DM as a Risk Factor for Mortality: Conflicting Data

Some conflicting data also exist. Harrison et al. observed a higher mortality in DM patients (unadjusted OR, 2.89; 95% CI, 2.56–3.26), but the association was attenuated after adjustment for age (29). In a smaller study from Brazil, also the presence of DM was not significantly associated with COVID-19 mortality in the multivariable analysis (94). According to an ICU study from Sweden, DM was not an independent risk factor for in ICU death (95). Observation based on very old individuals (age over 80 years old) also failed to identify DM as a risk factor for mortality after COVID-19 infection (96). Two meta-analysis as well reported a non-significant relationship among DM and mortality from COVID-19 infection (97, 98).

## 4 DM Patients’ Characteristics Associated With Severe Infection

### 4.1 The Role of DM-Related Complications on Disease Severity

In order to address risk factors related to worse outcome among DM patients, Cariou et al. contacted the CORONADO study, a multicenter study from France (99). They observed that among 1,317 patients with DM (T2DM 88.5%) that they examined, the primary outcome, namely, intubation and/or death within 7 days of admission, was independently and positively associated with body mass index (BMI), dyspnea, C-reactive protein (CRP), and aspartate aminotransferase (AST) and negatively associated with lymphocyte count (99). Age, treated obstructive sleep apnea, microvascular and macrovascular DM complications, dyspnea, elevated CRP or AST, low platelet count, or estimated glomerular filtration rate (eGFR) independently increased the risk of death on day 7 of hospitalization (99). On the contrary, glycated hemoglobulin (HbA1c), on admission plasma glucose, and the type of DM were not predictors of death and/or intubation in the multivariable analysis (99). In addition, in a sub-analysis of the CORONADO study focused on T1DM, older age was the only predictor of poor prognosis in the multivariable analysis (100).

### 4.2 Age, Sex, and Comorbidities as Risk Factors for Severe Disease Among Patients With DM

Holman et al. on the other hand, used UK national records and noticed that male sex, older age, renal or heart failure, history of stroke, non-white ethnicity, and socioeconomic deprivation were related to increased mortality among DM patients with COVID-19 infection (101). An HbA1c value ≥10% for type 1 DM (T1DM) and ≥7.6% for T2DM was also positively associated with mortality (HR, 2.23; 95% CI, 1.5–3.3; and HR, 1.22; 95% CI, 1.15–1.3 respectively) (101). A U-shaped relationship between BMI and risk of death was also observed; BMI <20 kg/m^2^ or >40 kg/m^2^ increased the risk for worse outcome compared with BMI of 25–29.9 kg/m^2^ for DM patients (101). Similar were the results of a large population based study from Scotland; among patients with DM, the risk of death or critical care unit admission was associated positively with age, male sex, HbA1c value, residence in a care home or deprived area, prior hospitalization in the past 5 years, prior smoking history, retinopathy, comorbidities, and the number of hypoglycemic drugs or other medication and negatively with systolic blood pressure, eGFR, and anti-hypertensive drug therapy (102). In this study, a j-shaped relationship was observed between COVID-19 severity and BMI (102). The type of DM and DM duration were not independent predictors of worse outcome (102). In addition, in a large cohort from Turkey, older age, male sex, obesity, pulmonary infiltrates on admission computer tomography, preadmission insulin therapy, and lymphopenia were independent predictors of death among T2DM patients with COVID-19 infection (59). On the contrary, HbA1c and blood glucose were not independently associated with mortality in the multivariable analysis (59).

### 4.3 Chronic Glycemic Control as Risk Factors for Severe Disease Among Patients With DM

Several other studies have also addressed the relationship between chronic glycemic control assessed by HbA1c value and COVID-19 infection outcome in patients with DM. A higher risk of death among patients with poorly controlled DM was reported by some authors (64, 103–106); however, the cutoff value of HbA1c for worse outcome was not consistent in the above studies. Williamson et al., in the OpenSAFELY study, observed that COVID-19-positive patients with DM and HbA1c values as low as 7.5% were at higher risk of death than those with HA1c <7.5% after adjustment for confounding factors (aHR, 1.95; 95% CI, 1.83–2.08 for HbA1c ≥7.5%; aHR, 1.31; 95% CI, 1.24–1.37 for HbA1c <7.5%, non-DM patients as reference group) (64). Moreover, in a study from China, not only high (≥6%) but also low (3%–4.9%) HbA1c values were related to higher mortality, perhaps due to higher prevalence of hypoglycemia among patients with low HbA1c (107). Other investigators, on the contrary, reported a no significant effect of preadmission HbA1c value on COVID-19 infection course (108–114). In a meta-analysis of eight studies, when regarded as a continuous value, HbA1c had a linear relationship with composite outcome, namely, death or COVID-19 infection worsening (p < 0.001), but not with mortality alone (p = 0.73); when however HbA1c was considered as a dichotomous value, poor glycemic control significantly increased the risk of death (p < 0.001) (115). In accordance were the results of a recent meta-analysis by Zhu et al. (116).

### 4.4 The Role of Glucose-Lowering Drugs on Disease Severity

Older age, male sex, comorbidities, and preadmission treatment with insulin were also reported as risk factors for poor prognosis after COVID-19 infection (56, 105, 108, 117–122). Confirmatory were the results of a meta-analysis including 22 studies (123). Preadmission DM medication may hence play a role in disease course among DM patients. Riahi et al. also observed that at-home use of insulin increased the risk of death among DM COVID-19-positive patients, independently of glycemic control and other confounding factors (124). On the contrary, at-home treatment with metformin was associated with lower mortality (23, 125–127), lower ICU (128), and hospital admission rates (125), and less complications, such as ARDS (125). In line were the results of a small meta-analysis, with a pooled OR of 0.59 for mortality among metformin users (95% CI, 0.43–0.79) (129). Interestingly, a large study with 27,493 T2DM patients reported a 30% lower risk of contracting COVID-19 among metformin users, although in-hospital mortality in case of infection was not affected by metformin treatment (130). Slightly lower mortality was also reported for sulfonylurea and glinides in two meta-analysis (131, 132). With regard to dipeptidyl peptidase 4 (DPP-4) inhibitors, most studies indicate a neutral (131, 132) or favorable effect on COVID-19 disease course (133, 134). In a large retrospective study by Emral et al. with 33,478 COVID-19-positive patients with T2DM, participants on treatment with DPP-4 inhibitors were at lower risk of dying compared with matched subjects on other antidiabetic drugs; however, no significant difference was observed in the risk for hospitalization or ICU admission between the two groups (135). In a meta-analysis, however, in-hospital but not preadmission use of DPP-4 inhibitors decreased the risk of death among selected COVID-19 patients with DM (136). Moreover, no significant impact of glucagon-like peptide-1 (GLP-1) analogues and sodium-glucose cotransporter 2 (SGLT2) inhibitors on COVID-19 outcomes was observed (131).

### 4.5 Laboratory Findings and COVID-19 Disease Severity

With regard to laboratory findings, elevated markers of inflammation (high CRP, white blood cell count, neutrophil count, ferritin, IL-6, and TNF-a), coagulation (such as d-dimers), and organ dysfunction (e.g., creatinine and lactate dehydrogenase), and low serum albumin or lymphocyte count were associated with worse outcomes among COVID-19-infected patients with DM in some studies (106, 109, 117, 137, 138). Likewise, in a meta-analysis by Dhar et al., elevated CRP, d-dimers, and low lymphocyte count were predictors of poor prognosis among DM COVID-19-positive subjects (139).

### 4.6 Body Weight as a Risk Factor for Severe Disease Among Patients With DM

Moreover, some studies assessed the link between BMI and COVID-19 infection course among DM subjects. Shukla et al. observed a j-shaped relationship with an increased risk among DM patients with BMI <18.5 or >35 kg/m^2^ compared with normal weighted subjects (122). Similar were the results of Mc Gurnaghan et al. as mentioned above (102). Other authors also reported a worse outcome for obese DM patients after COVID-19 infection (88, 99, 108).

In summary, age, male sex, comorbidities, DM microvascular or macrovascular complications, obesity or being underweight, and laboratory markers of inflammation, coagulation, and organ dysfunction are associated with worse outcomes among patients with DM and COVID-19 infection. Preadmission hypoglycemic agents also play a role in prognosis; metformin presents the more favorable profile, while insulin use may be a negative predictor. Poor chronic glycemic control, DM type, or duration may affect COVID-19 disease course, but there is no solid evidence. The role of acute hyperglycemia to COVID-19 infection deterioration will be discussed in detail below.

## 5 T1DM and COVID-19 Disease

The aforementioned studies either included patients with DM without any discrimination between T1DM and T2DM or focused on patients with T2DM, perhaps because of the low numbers of T1DM patients examined. There are, however, some data about patients with T1DM and COVID-19 infection in the literature. In a small ICU study, T2DM was independently associated with the need for intensive care (aOR, 2.47; 95% CI, 2.12–2.87), while T1DM was not (95). In accordance were the results reported by Dennis et al.; patients with DM and COVID-19 admitted to the hospital or ICU were at higher risk of death than patients without DM (aHR, 1.23; 95% CI, 1.14–1.32), but the relationship was not significant for patients with T1DM in subgroup analysis (aHR, 1.25; 95% CI, 0.86–1.84) (140). On the contrary, in a large study that included the whole population of Scotland, the risk of fatal or critical care unit treated COVID-19 infection was higher among T1DM (aOR, 2.39; 95% CI, 1.82–3.16) than T2DM patients (aOR, 1.37; 95% CI, 1.28–1.47) (102). The risk of death/critical disease was also higher for DM patients overall than for patients without DM (aOR, 1.39; 95% CI, 1.30–1.49) and had a reverse relationship with age among patients with DM (102) Similarly, in a whole-population survey from England, Barron et al. reported aORs for in-hospital mortality of 3.51 (95% CI, 3.16–3.9) and 2.03 (1.97–2.09) for T1DM and T2DM, respectively (141).

In conclusion, the relationship between T1DM and COVID-19 severity is less well established than that between T2DM and COVID-19 infection; several data, however, highlight that patients with T1DM are at increased risk of poor outcomes compared with patients without DM. Considering also the relatively younger age of patients with T1DM than that of T2DM patients, more research is needed to clarify whether DM type has an impact on COVID-19 disease course or not.

## 6 Stress Hyperglycemia, DM Hyperglycemia, Newly Diagnosed DM, and COVID-19 Infection

The American Diabetes Association (ADA) defines new-onset hyperglycemia without diabetes when fasting plasma glucose (FPG) is between 5.6 and 6.9 mmol/L and/or HbA1c is between 5.7% and 6.4%, in the absence of pre-existing dysglycemia (142). Newly diagnosed diabetes (NDD) is defined as two abnormal samples either FPG ≥7.0 mmol/L, HbA1c ≥6.5%, or a random glucose level ≥11.1 mmol/L with symptoms of hyperglycemia, without pre-existing diabetes (142). During acute illness, the release of hormones that counter-regulate insulin and an excess cytokine action may cause the so-called stress hyperglycemia, even in persons with formerly normal glucose metabolism (143). Here, we try to assess the importance of glucose levels for COVID-19 infection course.

In a study by Cai et al., fasting blood glucose (FBG) higher than 7 mmol/L was a predictor of in-hospital mortality among COVID-19-infected patients, independently of the history of DM (144). Interestingly, in subgroup analysis, FBG >7 mmol/L was a risk factor for death among non-DM patients (p < 0.001), but the association was not statistically significant for patients with DM (144). Similar were the results of another small study from Korea (145). Sun et al. also observed that FBG ≥7 mmol/L was independently associated with COVID-19 severity and mortality (60). In addition, among 184 COVID-19-hospitalized patients examined, mean FBG on admission was positively related to risk of intubation (146). Moreover, in two cohorts of non-DM patients hospitalized for COVID-19 infection, high FPG was an independent predictor of mortality (aOR, 3.54; 95% CI, 1.25–10.06 and aHR, 2.30; 95% CI, 1.49–3.55, respectively) (147, 148). Song et al. also examined COVID-19 subjects without DM; high FBG patterns, composited by at least three measurements during hospitalization, were related to higher risk of death (149). Of note, patients with decreasing trends of FBG due to hypoglycemic therapy were at lower risk compared with those with increasing FBG values over time (149).

Interestingly, Alahmad et al. tried to quantify the relationship between FBG and COVID-19 outcomes; they observed that FBG ≥7 mmol/L was associated with a 15-fold higher risk of ICU admission (OR, 14.57; 95% CI, 6.87–32.59), when FBG considered as a dichotomous variable and that 1 mmol/L rise in FBG increased the risk of ICU admission 1.59 times (95% CI, 1.38–1.89) in the linear model (150). Comparable were the results of the categorical model (150). Likewise Alshukry et al. noticed a 1.52 times higher risk of death (95% CI, 1.34–1.72) for each 1 mmol/L increase in FBG value (151). In consistence were the findings of a small study from China; fasting plasma glucose was related positively with mortality and ICU admission, with the risk being extremely high for glucose >11.1 mmol/l (HR, 11.55; 95% CI, 4.45–29.99) (152). Yuan et al., on the other hand, noticed that FBG values in the range of 3–4.9 mmol/L were related to the best prognosis (107).

Mean FBG over the first 7 days of hospitalization was also reported as an independent predictor of progression to severe disease (aRR, 2.09; 95% CI, 1.05–4.14), while 2 h postprandial blood glucose was not (111). Li et al. focused on DM patients and also noticed higher prevalence of death, in-hospital complications, and ICU admissions among DM patients with an admission glucose >11 mmol/L (153). High blood glucose during the first day of hospitalization or FBG on admission were also related with worse radiological findings (154, 155). Of note, in a study by Klonoff et al., admission hyperglycemia (glucose >250 mg/dl) was an independent predictor of mortality among patients directly admitted in the ICU (aHR, 3.14; 95% CI, 1.44–6.88; reference group glucose <140 mg/dl), but not for non-ICU hospitalized patients (156). For non-ICU patients, hyperglycemia on days 2–3 of hospitalization best correlated with mortality (aHR, 7.17; 95%CI, 2.62–19.62); hypoglycemia also increased the risk of death for both groups (OR, 2.2; 95% CI, 1.35–3.60) (156). Moreover, Wu et al. examined 2,041 hospitalized COVID-19 patients and divided them in two groups, namely, critical and non-critical (80). They observed that glucose on admission and median blood glucose were both independent predictors of mortality for critical cases and correlated with disease progression for non-critical cases (80).

In a UK cohort of 1,122 COVID-19 patients, DM, and/or uncontrolled hyperglycemia, defined as ≥2 blood glucoses measurements >10mmol/L within any 24-h period, were associated with higher mortality and prolonged hospitalization compared with non-DM/non-hyperglycemic patients (157). Patients with uncontrolled hyperglycemia without DM were also at higher risk than patients with DM (157). Likewise, in a small study from the USA, hyperglycemia, defined as glucose >11.1 mmol/L on admission, was a predictor of both mortality and complications among non-DM patients, while for patients with T2DM, elevated blood glucose was associated with ICU admission and AKI, but not with death (158). Similar were the results by Chen et al.; hyperglycemia (glucose ≥7.78 mmol/L) was a risk factor of mortality overall and among patients without DM, but the relationship was not significant among patients with DM (117). Zhang et al. also observed that hyperglycemia (defined as 2 or more glucose measurements ≥7.8 mmol/L) was related to high inflammatory markers and severe or critical disease, even after adjustment for confounding factors, while DM was not (159). Likewise, hyperglycemia was reported as the only independent predictor of COVID-19 infection course and related to 30% higher risk of death compared with patients with DM (160). Furthermore, based on findings from a small ICU cohort, authors noticed that mean blood glucose during ICU stay was independently related to mortality, in contrast with DM (161).

On the other hand, according to Leng et al., both glucose on admission and glucose variability were related to severe COVID-19 infection (162). Moreover, in a small cohort from Wuhan, glucose fluctuation and hyperglycemia during the first week of hospitalization, but not FBG on admission, were associated with increased mortality and COVID-19 complications among both DM and non-DM patients (163). Similarly, among DM patients, glucose fluctuation measured by a continuous glucose monitoring was associated with poor prognosis (164). Moreover, in a large study by Zhu et al., patients with DM and low glycemic variability (blood glucose, 3.9–10 mmol/L) were less likely to have COVID-19 complications or die compared with DM patients with poorly controlled blood glucose (65). With regard to random blood glucose (RBG), elevated values on admission were related to higher mortality, plasma levels of IL-6, and CRP (165), and increased severity of COVID-19 infection (166). According to other authors, however, RBG on admission was not significantly associated with disease prognosis (109, 113, 167, 168).

The above findings are summarized by some recent meta-analysis. In a meta-analysis of 16 studies by Yang et al., high blood glucose on admission (FBG or RBG) increased the risk of fatal (OR, 3.45; 95% CI, 2.26–5.26) and severe/critical (OR, 2.08; 95% CI, 1.45–2.99) disease among COVID-19-infected patients (169). However, no discrimination was made between DM and non-DM participants (169). According to another meta-analysis of 35 studies, elevated FBG on admission was independently associated to disease severity (risk ratio, 1.33; 95% CI, 1.26–1.40) with a 33% increase in risk of severe disease for each 1 mmol/L rise in FBG value (170). High FBG levels were also related to mortality (RR, 1.81; 95% CI, 1.41–2.33); the evidence was, however, weak. In addition, FBG was a better predictor of disease course among non-DM patients than those with DM (170). Of note, RBG was not independently related to COVID-19 infection outcomes (170). Moreover, Sachdeva et al. focused on patients without DM history and noticed higher rates of severe (OR, 1.837; 95% CI, 1.368–2.465) or fatal (OR, 2.822; 95% CI, 1.587–5.019) disease among hyperglycemic compared with normoglycemic individuals (171).

As it concerns NDD, Fadini et al. reported a significantly higher risk (p = 0.04) of ICU admission and/or death among patients with NDD (RR, 3.06; 95% CI, 2.04–4.57) than patients with pre-existing DM (RR, 1.55; 95% CI, 1.06–2.27), with non-DM patients being the reference group (172). High FPG on admission was also an independent predictor of severe COVID-19, with a 15% rise in the risk for each 2 mmol/L increase in FPG, and the association was stronger for non-DM subjects compared with DM (172). In accordance were the findings of a study from Mexico; patients with NDD were at higher risk for fatal COVID-19 infection (HR, 5.51; 95% CI, 1.28–23.81) than patients with known DM (HR, 4.98; 95% CI, 1.19–20.74) or pre-DM (HR, 3.35; 95% CI, 0.79–14.3) compared with non-DM subjects, even after adjustment for confounding factors (aHR, 5.50; 95% CI, 1.16–26.71) (173). An FBG value over 7.8 mmol/L was also an independent predictor of severity and mortality (173). A worse outcome among NDD compared to patients with known DM was also reported in a Chinese cohort of 2,880 COVID-19 patients (107). Likewise, NDD was related to higher mortality and in-hospital complications compared with both pre-existing DM and hyperglycemia without DM (174). As well, on admission, FBG correlated positively with the risk of death (174). On the contrary, in a sub-analysis of the CORONADO study, Cariou et al. observed a non-significant difference in mortality and COVID-19 complications between NDD and DM (175). Similarly, Liu et al. noticed that patients with DM overall were at higher risk of death than non-DM subjects, but there was no significant difference in mortality between patients with known DM and NDD (176).

The above evidence support high blood glucose as a predictor of poor prognosis regardless of the history of DM. Hence, a reasonable question is whether treatment of hyperglycemia can improve disease outcomes. Previous studies before COVID-19 pandemic failed to agree about the benefits of strict glucose control in critically ill hospitalized patients (177). In order to assess the effect of hypoglycemic treatment on COVID-19 infection course, Sardu et al. contacted a study including 59 COVID-19 hospitalized patients with initially moderate disease (178). Among hyperglycemic (defined as plasma glucose >7.7 mmol/L on admission), both DM and non-DM patients, reduction in blood glucose with insulin infusion reduced the risk for severe disease or death compared with those with hyperglycemia not treated with an insulin pump (178). More precisely, a 0.56 mmol/L reduction in blood glucose between day 1 and 18 was related to 11% reduction in the risk of severe outcome for hyperglycemic participants (178). Of note, hyperglycemia and DM were positively associated with poor outcome; patients with both hyperglycemia and DM were at higher risk, followed by hyperglycemia alone, DM without hyperglycemia, and normoglycemic/non-DM subjects (178). Likewise, acute reduction in blood glucose during the first 24 h of hospitalization was related to lower risk of severe disease or death among patients with hyperglycemia with or without DM (179).

In summary, hyperglycemia during COVID-19 disease course is associated with worse clinical outcomes. Glucose fluctuation and FBG seem to be better predictors than RBG measurements. In addition, hyperglycemia is a stronger risk factor for patients without DM than patients with known DM, reflecting perhaps infection burden and not glucose metabolism defects in previously normoglycemic individuals. Newly diagnosed DM may also be related to worse prognosis than pre-existing DM, and measures for lowering blood glucose could prevent disease progression.

## 7 Impact of COVID-19 on DM Course/Complications

An increase in admissions for diabetic ketoacidosis (DKA) was observed after the occurrence of COVID-19 pandemic compared with the previous years (180, 181). A turn towards T2DM and new-onset DM-related DKA was also observed, in contrast with pre-COVID-19 era, in which T1DM was more frequently reported among patients with DKA (180). Therefore, it is possible that COVID-19 infection is responsible for the observed increase in DKA incidence, either directly or indirectly. Moreover, in an effort to assess possible changes in DKA patients’ characteristics, Wallet et al. compared COVID-19-positive and COVID-19-negative subjects with DKA. The two groups did not differ in DKA severity; however, T2DM was more frequent in COVID-19-positive than COVID-19-negative DKA cases, and T1DM patients with COVID-19 had higher blood glucose than T1DM without COVID-19 (182). Shah et al. also assessed 82 cases of DKA or hyperglycemic hyperosmolar syndrome among COVID-19-positive and COVID-19-negative subjects; they observed a higher prevalence of T2DM among COVID-19-positive than COVID-19-negative individuals (183). Longer hospital stay and higher frequency of hypoglycemia were also reported among COVID-19-positive participants, although they experienced less severe acidosis (183). Hence, we could assume that COVID-19 infection may affect DM course. In addition, in a meta-analysis of 68 articles, 639 cases of DKA in patients with COVID-19 were investigated in order to picturize their profile; patients with T2DM had worse prognosis than T1DM (184). Older age, male sex, obesity, high blood glucose, and anion gap were also risk factors for increased mortality (184).

As discussed above, infection of b-cells by SARS-CoV-2 can lead to cell death and decrease in insulin release (177); excess inflammation may also increase insulin resistance in peripheral tissues (176). Thus, apart from complications in people with DM, it is also possible that COVID-19 triggers new-onset DM in formerly non-DM patients (185). Confirming were the findings from a meta-analysis of eight studies that reported a high prevalence of NDD among hospitalized COVID-19 patients (pooled proportion, 14.4%) (186).

On the other hand, an indirect effect of COVID-19 pandemic on glucose control is also possible; we could hypothesize that lockdown and consequent reduction in physical activity, mental stress, difficulties in access to health structures, and financial issues may impact DM control negatively. In a study from Japan, an increase in HbA1c was observed after COVID-19 pandemic outbreak (187). Likewise, in a small study with T1DM patients, deterioration in glucose control was noticed (188). Similar were the results from an Indian cohort (189). On the contrary, other studies did not report any deterioration in glucose parameters during lockdown (190–193).

**Figure 1:**
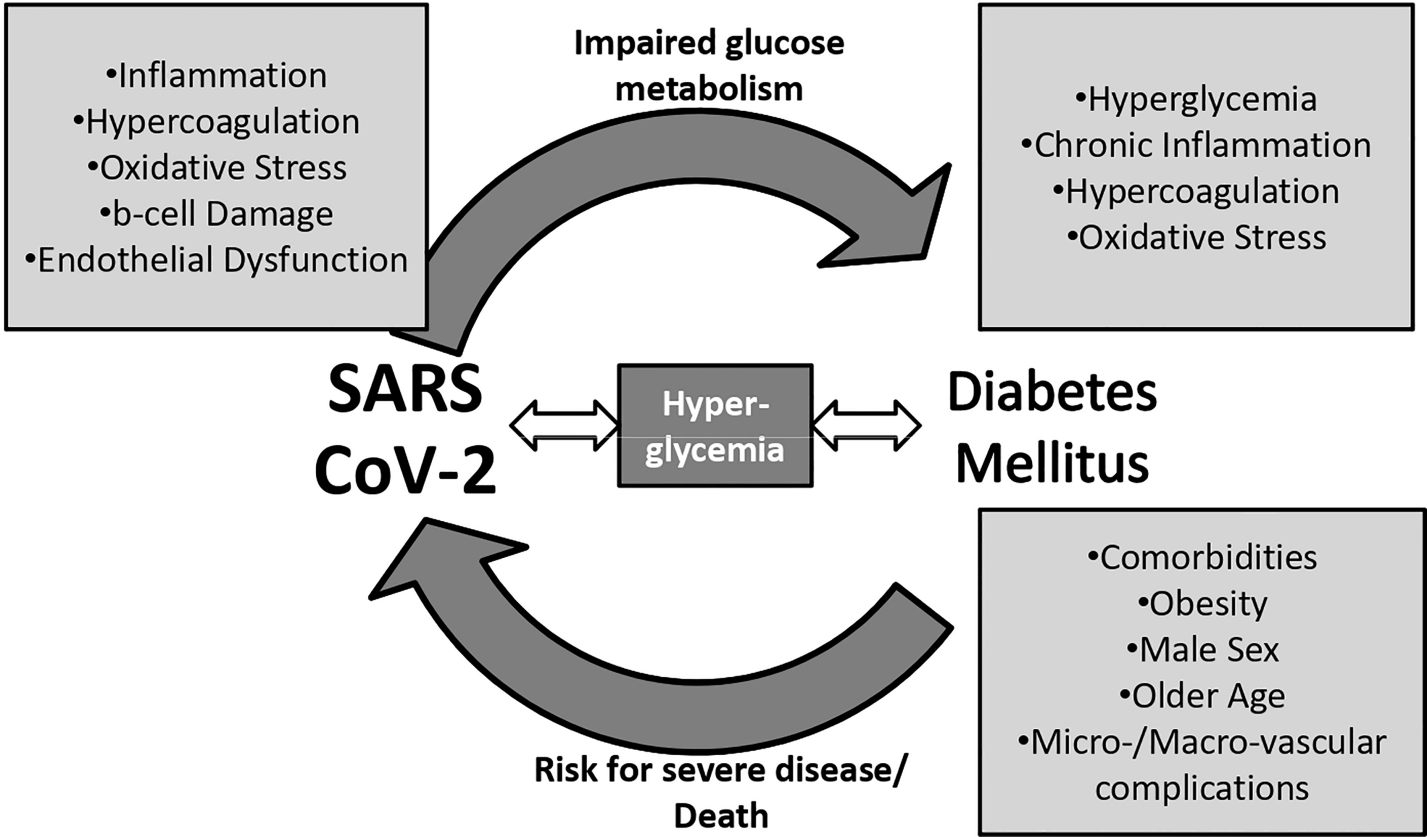
The vicious cycle between diabetes mellitus, hyperglycemia, and severe acute respiratory syndrome coronavirus 2 (SARS-CoV-2).

Putting all together, SARS-CoV-2 may deteriorate glucose control and trigger deregulation or even evolve DM among infected patients. Changes in everyday life due to pandemic could have also a negative impact on DM patients. Hence, the relationship between COVID-19 infection and DM is not straightforward; a vicious cycle may better explain the way SARS CoV-2 and DM affect each other. **Figure 1** below summarizes the interaction between COVID-19 infection and DM/ hyperglycemia.

## 8 Post-Discharge Implications

Post-COVID-19 or long COVID-19 syndrome refers to symptoms that persist for several weeks after the acute face of COVID-19 infection and cannot be explained elsewhere (194). Fatigue, loss of smell, chest pain, shortness of breath, and digestive and neurological problems, such as depression and headache, are the most frequent complains (195, 196). Patients with more severe disease during the acute phase are more likely to experience prolonged symptoms and residual lung dysfunction (196). Patients with DM are at higher risk for severe infection as discussed above; hence, we could assume that they are more prone to post-COVID-19 syndrome. Hence, according to a small study, the presence of DM increased the risk of persistent symptoms after recovery from COVID-19 infection (197). Similarly, in a cohort of 734 COVID-19-infected subjects, patients with DM reported pain and reduced mobility more frequently than non-DM individuals, 1 month after the COVID-19 acute infection was resolved (p < 0.05) (198). No difference was, however, noticed in the incidence of other post-COVID-19 symptoms (198). In addition, the prevalence of T2DM among patients with post-COVID-19 syndrome was estimated at 15% according to a recent meta-analysis (195), which was higher than the prevalence of DM in the general population (36). In three other studies, however, patients with DM were not more symptomatic than patients without DM during post-COVID-19 period (199–201).

Other post-discharge complications constituted the need for readmission, and death. Kingery et al. observed higher readmission rates among patients with DM (HR, 1.54; 95% CI, 1.06–2.23) compared with non-DM subjects during a 30-day follow-up period after recovery from COVID-19 infection and hospital discharge (202). In addition, DM was associated with increased mortality during the first month post-discharge (HR, 1.98; 95% CI, 0.99–3.99) in the univariable analysis, but was attenuated after adjustment for confounding factors (202). Lavery et al. also reported higher risk for readmission among patients with DM compared with non-DM individuals (OR, 1.21; 95% CI, 1.14–1.28) (203). Similar were the results of two other studies (204, 205). Likewise, the composite outcome readmission and/or death occurred more frequently among patients with DM during the post-COVID infection period (HR, 1.71; 95% CI, 1.17–2.52) (206). De Lorenzo et al. also estimated that history of DM, age, long of hospital stay, and non-invasive ventilation predicted the need for close follow-up post-discharge (207). Moreover, the risk for severe COVID-19 reinfection was reported to be higher among patients with DM (RR, 1.22; 95% CI, 1.07–1.38) (208). On the contrary, no association between DM and rehospitalization was observed in a retrospective study from New York City (209).

Moreover, after the COVID-19 pandemic outbreak, a rise in cases of mucormycosis was observed, and a new entity was described, COVID-19-associated mucormycosis (CAM) (210). The use of corticosteroids in the treatment of COVID-19 infection (211), changes in the iron metabolism and immune system responses, and endotheliitis caused by the virus itself (12) may predominate vulnerable individuals to mucor infections (212). Diabetes mellitus is also a well-documented risk factor for mucormycosis (213). In a large Indian cohort of 2,826 COVID-19 patients with mucormycosis, 71% were male, 87% received corticosteroids, 78% had history of DM, and 44% had uncontrolled DM or DKA (214). Although most cases of CAM were identified during the first 2 weeks of COVID-19 infection, in 44% of patients, the fungal infection was diagnosed 14 days or more after COVID-19 diagnosis (214). In line were the results of a recent review of 30 CAM cases (215). Likewise, in a pooled analysis of 28 articles, 40.6% of mucormycosis cases were diagnosed after recovery from COVID-19 infection (216). Male sex was also predominant, while 80% of patients had DM history and 14% simultaneous DKA (216). Physicians should, hence, be aware of symptoms of mucormycosis and follow-up high-risk patients, such as males patients with poorly controlled DM or a recent episode of DKA.

## 9 Conclusions

In summary, patients with DM are more likely to experience severe or fatal COVID-19 infection than patients without DM and need hospitalization or ICU admission. Age, male sex, comorbidities, DM complications, obesity or low body weight, and laboratory markers of inflammation or coagulation increase the risk for unfavorable outcomes among DM patients. Acute hyperglycemia defined as high FBG or glucose fluctuation, rather than HbA1c, are also predictors of poor prognosis, especially in patients without pre-existing DM. Newly diagnosed DM also plays an important role in disease course. Measures for COVID-19 contracting prevention, vaccination for SARS-CoV-2, early diagnosis after infection, and close follow-up, and better glycemic control should be priorities for physicians when treating patients with DM. Post-discharge complications, such as post-COVID syndrome, readmission or mucormycosis may also be more frequent among patients with DM, and special attention should be paid. In the figure below, we summarize the main findings of our review.

## Author Contributions

ET and ES conducted literature search. ET, ES, CS, AT, and NT composed the review. The manuscript was critically reviewed by NT. All authors contributed to the article and approved the submitted version.

## Conflict of Interest

The authors declare that the research was conducted in the absence of any commercial or financial relationships that could be construed as a potential conflict of interest.

## Publisher’s Note

All claims expressed in this article are solely those of the authors and do not necessarily represent those of their affiliated organizations, or those of the publisher, the editors and the reviewers. Any product that may be evaluated in this article, or claim that may be made by its manufacturer, is not guaranteed or endorsed by the publisher.
